# Genomic prediction when some animals are not genotyped

**DOI:** 10.1186/1297-9686-42-2

**Published:** 2010-01-27

**Authors:** Ole F Christensen, Mogens S Lund

**Affiliations:** 1 Aarhus University, Faculty of Agricultural Sciences, Dept of Genetics and Biotechnology, Blichers Allé 20, PO BOX 50, DK-8830 Tjele, Denmark

## Abstract

**Background:**

The use of genomic selection in breeding programs may increase the rate of genetic improvement, reduce the generation time, and provide higher accuracy of estimated breeding values (EBVs). A number of different methods have been developed for genomic prediction of breeding values, but many of them assume that all animals have been genotyped. In practice, not all animals are genotyped, and the methods have to be adapted to this situation.

**Results:**

In this paper we provide an extension of a linear mixed model method for genomic prediction to the situation with non-genotyped animals. The model specifies that a breeding value is the sum of a genomic and a polygenic genetic random effect, where genomic genetic random effects are correlated with a genomic relationship matrix constructed from markers and the polygenic genetic random effects are correlated with the usual relationship matrix. The extension of the model to non-genotyped animals is made by using the pedigree to derive an extension of the genomic relationship matrix to non-genotyped animals. As a result, in the extended model the estimated breeding values are obtained by blending the information used to compute traditional EBVs and the information used to compute purely genomic EBVs. Parameters in the model are estimated using average information REML and estimated breeding values are best linear unbiased predictions (BLUPs). The method is illustrated using a simulated data set.

**Conclusions:**

The extension of the method to non-genotyped animals presented in this paper makes it possible to integrate all the genomic, pedigree and phenotype information into a one-step procedure for genomic prediction. Such a one-step procedure results in more accurate estimated breeding values and has the potential to become the standard tool for genomic prediction of breeding values in future practical evaluations in pig and cattle breeding.

## Background

Genomic selection [1] has become the new paradigm in animal breeding programs using marker-assisted selection. It may increase the rate of genetic improvement, reduce the generation time, and provide higher accuracy of estimated breeding values (EBVs). Genomic prediction of breeding values can be based on a linear mixed model using matrix computations or a non-linear mixture type of model using Markov chain Monte Carlo (McMC) procedures. In this paper we provide a natural extension of a linear mixed model to the situation with non-genotyped animals.

A marker-based relationship matrix has been used by a number of authors, in particular VanRaden in [2] and [3], but also Gianola and van Kamm [4] in a dual formulation of their model. The types of genomic relationship matrices studied here are on the form(1)

as in VanRaden [3], but other types of genomic relationship matrices are discussed in the discussion section. In VanRaden [3] it is assumed that all animals are genotyped, which is unlikely to be a common scenario. In particular, in pig breeding it is probable that only boars or other selection candidates are genotyped, and in cattle breeding, traits being recorded for millions of animals it is very unlikely that all will be genotyped. We present an extension of matrix (1) in the situation where not all animals are genotyped. The approach presented here combines the relationship matrix (1) with a model for the markers. By marginalisation of the markers of non-genotyped animals a natural extension of (1) is obtained. The resulting extension of the genomic relationship matrix is the same as the one derived in Legarra et al. [5], but the details in the derivation are somewhat different and the derivation therefore sheds more light on this result.

To capture genetic variation not associated to the markers in a given SNP-panel, the model can also contain a polygenic genetic effect with the usual pedigree derived additive relationship matrix, as considered by [4,6] among others. The extension of the genomic relationship matrix to non-genotyped animals together with the addition of the polygenic effect provide a natural one-step procedure to blend the information from relatives and the genomic information into a combined genomically enhanced breeding value (GEBV). Genomic prediction with both a polygenic effect and with incomplete genotyping has been considered by a number of authors. Using a joint model for phenotypes and markers and using Bayesian inference, a general solution to sample missing markers in each McMC iteration has been suggested [4,7]. However, with a large number of SNP markers and many animals without genotypes such a solution seems computationally unfeasible in practice. In Gianola et al. [7] bivariate models are suggested, where the two traits are the traits of the genotyped and non-genotyped animals, respectively, and the genetic effect for a genotyped animal is the sum of a polygenic effect and a genomic effect whereas the genetic effect for a non-genotyped animal is just a polygenic effect (correlated with the polygenic effect of the genotyped animals). Since the model does not contain a genomic genetic effect for the non-genotyped animals, the phenotypic information from non-genotyped animals closely related to a given genotyped animal does not propagate properly into the estimate of the genomic genetic effect for this animal. Alternatively, the approach by Baruch and Weller [8] involves several steps, where first, expected genotypes are computed for non-genotyped animals, then marker effects are estimated (using expected genotypes for non-genotyped animals), phenotypes are adjusted by known or expected marker effects, and finally polygenic EBVs are computed from adjusted phenotypes. Although somewhat similar in idea to the approach taken here, the approach in [8] does not propagate any uncertainty from one step in the procedure to the next step, and the effects are not estimated simultaneously.

## Methods

We assume that markers are summarised into a gene content matrix, *m *(*m*_*ij *_= -1, when the SNP *j *of individual *i *is 11, *m*_*ij *_= 0 for 12, and *m*_*ij *_= 1 for 22), and we use capital letters *M*_*ij *_to denote when the markers are random variables. For the genomic relationship matrix (1), the matrix *p *is the expectation of *M*, i.e. the entries in column *j *are *p*_*j *_= 2(*ρ*_*j *_- 1/2) with *ρ*_*j *_being the allele frequency of the second allele at loci *j*, and *h *is a diagonal matrix chosen such that E[*G*(*M*)] = *A*, the usual pedigree derived additive relationship matrix. In VanRaden [3] three different genomic relationship matrices are presented, where the first two are on the form in (1), and here, we focus on the first one(2)

with *s *= ∑_*j *_2*ρ*_*j*_(1 - *ρ*_*j*_).

The model is as follows(3)

where *y *is phenotype, *X *and *Z *are incidence matrices, *β *denotes fixed effects, *e *is error,  is the polygenic genetic effect, and  is the genomic genetic effect. Here *A *is the usual pedigree derived additive relationship matrix, and G*(*m*^*obs*^) is the extension of (2) to be derived in the following section.

In the following sections, first, we derive the extension of the marker based relationship matrix, G*(*m*^*obs*^), and second, we study the variance-covariance matrix of the combined genetic effect *g *+ *a*. Then procedures for parameter estimation using AI-REML, and breeding value estimation are presented. Finally, a simulation data set is described.

### Genomic relationship matrix with a relationship of markers

Gengler et al. [9] suggested that missing genotypes could be modelled using the usual mixed model methodology with relationship matrix *A*. We now combine that idea with the genomic relationship matrix on the form (1). For simplicity, the derivation is made for the form (2), but it is straight-forward to generalise to (1) also.

The model for the genomic genetic effect is as follows

where *M *is the gene content matrix. We assume that E[*M*_*j*_] = 1*p*_*j*_, Var(*M*_*j*_) = *v*_*j*_*A*, with *A *the usual relationship matrix, *v*_*j *_= 2*ρ*_*j*_(1 - *ρ*_*j*_), and *s *= ∑_*j *_*v*_*j*_. The covariances of *M*_*j*_, and *M*_*j' *_for two different loci *j *≠ *j' *are on the form Cov(*M*_*j*_, *M*_*j'*_) = *v*_*j*,*j' *_*A *where the *v*_*j*,*j' *_s are unspecified since they are cancelling in the derivations that follow.

We split *M *into two sub-matrices containing the animals with observed genotypes and those without, respectively,

and in the following we distinguish between small letter *m*^*obs*^ (observed realisation of random variables *M*^*obs*^) and capital letter *M*^*miss *^(unobserved markers are random variables). In Appendix A, the mean vector and variance-covariance matrix of the conditional distribution [*g*|*m*^*obs*^] (with *M*^*miss *^marginalised out) are shown to be

Where(4)

When all animals have been genotyped, G*(*m*^*obs*^) = *G*(*m*^*obs*^), and when no animals have been genotyped, G*(*m*^*obs*^) = *A*, which makes the extension in (4) rather elegant. We assume that the distribution of [*g*|*m*^*obs*^] is multivariate normal, which for the non-genotyped animals is not strictly true, but an approximation.

The inverse of the genomic relationship matrix may be obtained from the inverse of *A*,(5)

Using some algebra, the inverse of the genomic relationship matrix becomes(6)

Considering the terms in (6), because of the low dimension of *G*(*m*^*obs*^) and *A*_11 _a direct inversion of these matrices should be possible for practical computations, and *A*^-1 ^is a sparse matrix which can be computed directly without constructing *A *itself and using standard techniques. To compute *A*_11 _there might be cases where most of the *A *matrix has to be computed, potentially causing a memory storage problem.

Alternatively, *A*_11 _= ((*A*^-1^)^-1^)_11 _may be computed using the formula (5) on *A*^-1 ^and using sparse matrix computation. The formula (6) requires that *G*(*m*^*obs*^) is invertible which may not actually be the case. In the next section this problem is automatically solved by combining the genomic genetic effect *g *with the polygenic effect *a*.

We also note that the determinant equals

where *A*_22 _- *A*_21 _*A*_12 _is easily obtained from *A*^-1^, and the determinant can be computed using sparse matrix computation.

### The combined genetic effect

The combined genetic effect is the sum of the genomic genetic effect and the polygenic effect,  = g + a, and using this notation the model (3) may now be written as(7)

where . Introducing the notation  and , then

with  = (1 - *w*)G*(*m*^*obs*^) + *wA*. Substituting (4) and rearranging the terms, we obtain

where

The parameter *w *is interpreted as the relative weight on the polygenic effect, and it may be estimated from data as shown in the next section or be chosen to equal a small value.

Similar to the previous section the inverse equals(8)

and here *G*_*w *_is necessarily invertible when *w *> 0 (even when *G*(*m*^*obs*^) is singular).

### Variance component estimation

Here we consider parameter estimation using average information (AI)-REML based on the mixed model equations [10,11](9)

where . We will not enter into details, but just note that the sparse structure of the left hand side matrix in (9) is the cornerstone for the fast computation of the AI-matrix used in the numerical maximisation of the REML likelihood. Considering the terms in this matrix, then *Z*^T^*Z *is a sparse matrix, and from (4) we see that  has some sparse structure, although  is a dense matrix. Depending on the proportion of animals genotyped it may in some cases not be necessarily advantageous to compute the AI-matrix using (9), but instead an AI-REML algorithm based on the inverse phenotypic variance-covariance matrix, , could be used, see [12]. Here, we assume that the majority of animals are not genotyped and use the sparse structure of G*(*m*^*obs*^)^-1 ^for AI-REML based on the mixed model equations.

The AI-REML method based on the mixed model equations is implemented in software DMU [13] and requires input in the form of the vector of phenotypes, the nonzero entries of  and the log-determinant log(det()) = log(det(*G*_*w*_)) + log(det(*A*_22 _- *A*_21_*A*_12_)). For a given *w *the software provides estimates of  and , values of the REML log-likelihood at the maximum and (when required) BLUE solution  and BLUP solution . Here, the parameter *w *is estimated by using a grid of values, i.e. *w *= 0.01, 0.03, ..., 0.19, and computing the REML log-likelihood for each value. The resulting profile likelihood curve, log , has a peak at the estimate , and a measure of the associated uncertainty is the interval {*w*|log  > log  - 3.84} where 3.84 is the 95% quantile of a *χ*^2^(1)-distribution.

### Breeding value estimation

Here we consider estimation (prediction) of breeding values. For animals included in the parameter estimation (animals with phenotypes, and some additional animals whose markers provide information about the unknown markers for non-genotyped animals with phenotypes), the GEBVs are the solution vector  to (9) with the parameter values being the estimated ones from the previous section. The software DMU provides these GEBVs and their precision.

For animals not included in the parameter estimation, then denoting this subset of animals by index 3 the GEBVs  are obtained by solving

where , *Z*_*all *_and  now contain all animals. Again software DMU provides these GEBVs and their precision.

For a scenario with a large number of genotyped animals whose marker information does not provide information for the parameter estimation, Appendix B presents a method for breeding value estimation where only part of the  needs to be computed.

### A simulated data set

The simulated data set is inspired by a pig nucleus breeding program, but is formulated in a simplified form. We assume, 10 chromosomes each 160 cM long, and a panel of *p *= 5000 equidistant SNP markers is used. It is assumed that 500 QTLs affect the phenotype, and the size of these effects is simulated from a Gamma(5.4, 0.42)-distribution. First, a base population consisting of 150 boars and 1500 sows is generated by assuming random mating for 50 generations in a population with an effective population size of 100. Then the following mating and selection scheme is followed for five generations. In each generation, 150 boars are mated with 1500 sows to produce 15000 offspring (half of them males). For the next generation, the 150 boars with the highest value of their own phenotype are selected, and 1500 sows are selected randomly. It is assumed that family records are available for all five generations, phenotypes of all boars available for all five generations (35000 records), and the selected boars in the last three generations are genotyped (450 animals). In addition, to estimate the allele frequencies required for the method, the 150 boars in the base population are genotyped (and the allele frequencies used are the estimated frequencies from these 150 boars). For prediction, it is assumed that 300 selection candidates (without phenotypes) for generation 6 are genotyped.

To evaluate the method advocated in this paper (one-step), two other methods are investigated. The first method (ped) computes traditional EBVs using the pedigree based relationship matrix (without using markers). The second method (two-step) is a two-step procedure similar to methods used in practical genomic selection [14,15] and is based on genotyped animals only using the model(10)

where *y*_*EBV *_is the vector of traditional EBVs, and  with *G*_*w *_= 0.99*G*(*m*^*obs*^) + 0.01*A*_11_.

For the one-step method, the genotypes of the selection candidates provide information about the genotypes of their (non-genotyped) mothers and hence information about other non-genotyped animals further back in the pedigree. Therefore they also provide some information about the genotypes of the boars without offspring, and since these boars have phenotypes but not genotypes then the selection candidates should be included in the parameter estimation. However, to investigate how important it is to include these animals, a second analysis (one-step-2) is also performed where they are not included. Finally, to investigate the importance of obtaining the allele frequencies in the base population, the scenario where the boars in the base population have not been genotyped is also studied. The use of three different allele frequencies are compared: 1) true allele frequencies (obtained from the 150 boars in the base population), 2) estimated allele frequencies for boars used in generation 3, 3) allele frequencies estimated using the approach by Gengler et al. [9].

## Results

**Figure 1:**
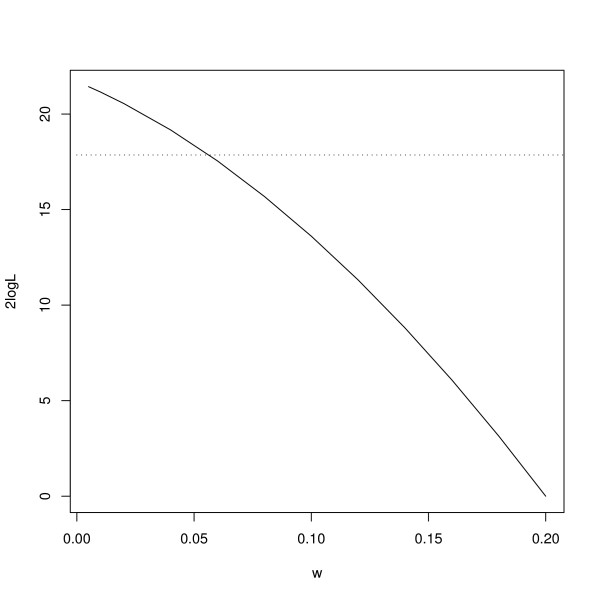
**The profile log-likelihood curve for *w***. The dotted line corresponds to a the 95% quantile for a *χ*^2^(1) distribution, and provides a 5% confidence interval of [0; 0.06] for *w*.

For the one-step method, the profile likelihood curve for *w *is shown in Figure 1. It is seen that the data do not support a large polygenic effect, with the estimate being about zero and the 5% confidence interval being about [0; 0.06]. For computational reasons, we decided to use  = 0.01.

The parameter estimates and the correlation between GEBVs and true breeding values (BVs) are shown in Table 1. For comparison, the prediction using the pedigree based relationship matrix (ped method) and the genomic prediction using (10) based on genotyped animals (two-step) are also shown. We observe that the two methods using a marker-based relationship matrix perform better than the method using the pedigree based relationship matrix, but as expected the one-step method performs the best.

**Table 1:** Results from model with  = 0.01.

Method			Cor. true BV
one-step	4.16	16.22	0.6598
ped	5.03	15.80	0.3537
two-step	7.56	0.069	0.5869
one-step-2	5.98	15.58	0.6596

Method one-step is the method advocated in this paper, method ped uses the pedigree based relationship matrix, and method two-step is the genomic prediction method using only genotyped animals (note that parameter estimates for this method cannot be compared to parameter estimates from the other two methods). Finally, one-step-2 differs from one-step in that it ignores the markers of selection candidates in the parameter estimation. The right-most column shows the correlation between the estimated and the true breeding value (BV).

Column four in Table 1 shows the result obtained when ignoring the genotypes of the 300 selection candidates in the parameter estimation (one-step-2). Even though the parameter estimates are somewhat different betweeen one-step and one-step-2, only a minor difference in the correlation between GEBVs and the true breeding values is seen. Hence, for this data set this specific computational short-cut performs well. Finally, the results from the analyses where the boars in the base population are not genotyped show that the choice of allele frequencies is very important for parameter estimation. When using the true allele frequencies,  ≈ 0 is obtained, whereas when using allele frequencies estimated from the observed genotypes,  = 1 is obtained for both methods estimating the allele frequencies. Since  = 1 corresponds to the usual animal model, no further results from this comparison are shown here. We conclude that for this data set the parameter estimation is sensitive to the allele frequencies used in the one-step method.

## Discussion

For genomic prediction an extension of a linear mixed model to non-genotyped animals has been derived here. The extension of the method makes it possible to integrate in an optimal way the genomic, pedigree and phenotype information into a one-step procedure for breeding value estimation. Due to the simplicity of the method, the fact that it extends the traditional breeding value estimation method in a natural way, and the possibilities of handling large populations, such a one-step procedure has the potential to become the standard tool for genomic prediction of breeding values in practical pig or cattle evaluations in the future. The practical implementation of the approach uses an existing software DMU, and therefore the approach can be easily extended to other types of models implemented by that software, in particular multivariate analysis and generalised linear mixed models.

For such a one-step procedure to become the standard tool for computing GEBVs in practical pig or cattle evaluations, some technical issues of the method need further development. First, computing times necessary for the construction and the inversion of *G*(*m*^*obs*^) are proportional to *p *and , respectively. These computations seem to be the computational bottle-necks for the method, and for a very large number of genotyped animals the method may not be feasible. Further research on efficient computation of *G*(*m*^*obs*^)^-1 ^seems necessary. Second, some computational short-cuts in the method could be imagined, as illustrated in our results by the good performance of the one-step method even when the marker information from selection candidates is ignored in the parameter estimation. Investigations by extensive simulation studies may reveal the benefits of other potential short-cuts. Third, the allele frequencies in the base population are considered known, or at least easily accessible. As illustrated in the results, the parameter estimation seems to be sensitive to the choice of these allele frequencies in a scenario with selection and where the base population itself has not been genotyped. To investigate whether the problems may be related to the strong selection on phenotype for the simulation data set, this analysis was repeated for a simulation with boars selected randomly. Here more sensible parameter estimates were obtained in the sense that  ≈ 0 when allele frequencies were estimated from observed genotypes. For practical dairy cattle evaluations, Misztal et al. [16] investigated the use of a number of different allele frequencies and obtained the best results by using *ρ*_*j *_= 1/2 for all *j *but replacing *s *= 2∑_*j*_*ρ*_*j*_(1 - *ρ*_*j*_) = *p*/2 with a another scaling *s *which in practice was larger than *p*/2. Of course, whether that result is due to selection in this real data set is not known. Further research on the effect of selection and on how to handle appropriately the issue with allele frequencies is needed.

An assumption behind the genomic relationship matrix (2) is that all regions of the genome are equally important for the trait of interest. It is possible to instead use *G*(*m*) ∝ (*m *- *p*)*h*(*m *- *p*)^*T *^where *h *is a diagonal matrix with known weights *h*_*jj *_=  with *b*_*j*_s being estimated SNP effects (estimated using for example a non-linear mixture type of model as in [1]). However, incorporating uncertainty on such estimated SNP effects into the method seems less straight-forward.

Considering other types of marker based relationship matrices, then(11)

with correlation parameter *ϕ*, corresponds to the method in [4] in it's dual formulation as a linear mixed model. For this choice of marker-based relationship matrix, the derivation of K*(*m*^*obs*^) = Var [*g*|*m*^*obs*^] is also possible, but as shown in Appendix C the form of the result differs from (4) in a number of ways. The implication is that using (4) and (6) with a marker based relationship matrix defined by (11) is possible, but lacks theoretical justification.

## Competing interests

The authors declare that they have no competing interests.

## Authors' contributions

OFC derived and implemented the methods, created and analysed the simulation study, and wrote the paper. MSL conceived the study, took part in discussions, and provided input to the writing of the paper. Both authors have read and approved the paper.

## Appendix A

Here the mean and variances of the conditional distribution [*g *| *m*^*obs*^] (with *M*^*miss *^marginalised out) are derived using formulas for conditional expectations, variances and covariances.

The mean vector

and the variance-covariance matrix

where

and

with

and subdivision corresponding to (*M*^*obs*^, *M*^*miss*^). Using that ∑_*j *_*v*_*j *_= *s*, we obtain Var [*g *| *m*^*obs*^] = G*(*m*^*obs*^) where

In the calculations above it is assumed that the conditional mean  and the conditional variance-covariance , and this is correct since

when Var(*M*) = *V *⊗ *A*.

In the main text we assume

where G*(*m*^*obs*^) is defined in (4). However, this is not strictly correct for a non-genotyped animal *i *where *g*_*i *_| *X *~*N *(0, *X*) with *X *here being a random variable with distribution [∑_*j *_(*M*_*ij *_- *p*_*j*_)^2^|*m*^*obs*^]. This conditional distribution will never lead to a marginal normal distribution for *g*_*i *_(the only exception is when *X *is a constant). The normal distribution of *g*|*m*^*obs *^is therefore only an approximation.

## Appendix B

In some scenarios the number of genotyped animals not included in the parameter estimation may be large, for example if phenotypes are expensive to obtain and therefore only observed on a small subset of the population. To reduce the computational burden of creating the whole (*m*^*obs*,*other*^) for all animals, a procedure is presented where only a part of this matrix needs to be computed.

For genotyped animals used in the parameter estimation, let  be the corresponding sub-vector of . Estimated breeding values of other genotyped animals not included in the parameter estimation (denoting this subset of animals by index 3) are obtained by

Where , and  and *A*_31 _= (*A*_*all*_)_31 _are sub-matrices of the full (containing all animals) genomic and polygenic relationship matrix, respectively. The matrices with index 32 are similarly defined. Since *m*^*other*^ does not influence *M*^*miss *^directly,

Considering the polygenic effect, then the assumption that *m*^*other *^does not influence *M*^*miss *^is equivalent to *A*_32 _- *A*_31_*A*_12 _= 0. Using this relation we obtain

Hence,

and therefore by using (8) and (5) the following form is obtained(12)

This shows that the GEBVs of such genotyped animals only depend on . It also shows that only a part of the full genomic relationship matrix for genotyped animals is necessary to compute, since *G*_*w*,33 _= (1 - *w*)*G*(*m*^*other*^) + *wA*_33 _does not enter into (12).

In some cases the matrix *A*_31 _may be prohibitive to compute directly due to a large number of animals. In such a case, , where  is computed directly and  may be obtained as the solution to the sparse system of equations

where (*A*_*all*_)^-1 ^is sparse and is computed directly, and  and  are dummy variables.

## Appendix C

Here follows the derivation of the extension of the marker-based relationship matrix

to non-genotyped animals.

The extension of the genomic relationship matrix is

As written in the discussion, the form of this matrix differs from (4) in a number of ways. First, all diagonal elements K*(*m*^*obs*^)_*ii *_= 1, and hence K*(*m*^*obs*^) does not simplify to the *A *matrix when no animals are genotyped. Second, the resulting matrix depends on the off-diagonal elements *v*_*jj*' _of *V*, since for non-genotyped animals *i *and *i' *the derivation

requires that *M*^1^,..., *M*^*p *^are statistically independent (implying that *V *is a diagonal matrix). Third, the conditional expectation  depends on the distributional assumptions of the model for *M*, not just first and second moments. Fourth, assuming a multivariate normal distribution of *M*, then

with  and  where these expectations and variances can be computed from the conditional expectations and variances given in Appendix A. The form exp(-*v*^2^/(1 + *τ*^2^))/ with the variance *τ*^2 ^occurring in two places, implies that that the elements in K*(*m*^*obs*^) cannot be expressed in matrix form as in (4) but are on a more complicated form.
